# Projected future climatic forcing on the global distribution of vegetation types

**DOI:** 10.1098/rstb.2023.0011

**Published:** 2024-05-27

**Authors:** Bethany J. Allen, Daniel J. Hill, Ariane M. Burke, Michael Clark, Robert Marchant, Lindsay C. Stringer, David R. Williams, Christopher Lyon

**Affiliations:** ^1^ Department of Biosystems Science and Engineering, ETH Zurich, Basel 4056, Switzerland; ^2^ Computational Evolution Group, Swiss Institute of Bioinformatics, Lausanne 1015, Switzerland; ^3^ School of Earth and Environment, University of Leeds, Leeds, LS2 9JT, UK; ^4^ Département d'Anthropologie, Université de Montréal, Montréal, Quebec, H3C 3J7, Canada; ^5^ Smith School of Enterprise and the Environment, University of Oxford, Oxford, OX1 3QY, UK; ^6^ Oxford Martin School, University of Oxford, Oxford, OX1 3BD, UK; ^7^ Department of Biology, University of Oxford, Oxford, OX1 3RB, UK; ^8^ Department of Environment and Geography, University of York, York, YO10 5NG, UK; ^9^ Leverhulme Centre for Anthropocene Biodiversity, University of York, York, YO10 5DD, UK; ^10^ York Environmental Sustainability Institute, University of York, York, YO10 5DD, UK

**Keywords:** climate change, global warming, biosphere, biome, biodiversity, anthropogenic

## Abstract

Most emissions scenarios suggest temperature and precipitation regimes will change dramatically across the globe over the next 500 years. These changes will have large impacts on the biosphere, with species forced to migrate to follow their preferred environmental conditions, therefore moving and fragmenting ecosystems. However, most projections of the impacts of climate change only reach 2100, limiting our understanding of the temporal scope of climate impacts, and potentially impeding suitable adaptive action. To address this data gap, we model future climate change every 20 years from 2000 to 2500 CE, under different CO_2_ emissions scenarios, using a general circulation model. We then apply a biome model to these modelled climate futures, to investigate shifts in climatic forcing on vegetation worldwide, the feasibility of the migration required to enact these modelled vegetation changes, and potential overlap with human land use based on modern-day anthromes. Under a business-as-usual scenario, up to 40% of terrestrial area is expected to be suited to a different biome by 2500. Cold-adapted biomes, particularly boreal forest and dry tundra, are predicted to experience the greatest losses of suitable area. Without mitigation, these changes could have severe consequences both for global biodiversity and the provision of ecosystem services.

This article is part of the theme issue ‘Ecological novelty and planetary stewardship: biodiversity dynamics in a transforming biosphere’.

## 1. Introduction

Biomes represent a well-established scheme for categorizing ecosystems, and particularly the vegetation within them, at the global scale. Biomes have previously been defined in myriad ways [1–5], but are broadly recognized as structurally and functionally (but not necessarily taxonomically) consistent plant communities, sometimes associated with a specific set of climatic conditions and soil states [2,3,6,7]. Animal communities are also typically assumed to be well described by these delineations (e.g. [3,5]), and in some cases can also play a role in determining biome composition, structure and distribution [8]. Continental drift and climate change have governed the shifting of biomes over geological timescales, as evidenced in the fossil record (e.g. [9–11]).

Humans have also shaped, and will continue to shape, the distribution of biomes across the world. Around three-quarters of terrestrial area has been populated for at least 12 000 years [12], with accompanying changes in flora and fauna, and the creation of anthropogenic biomes (anthromes) [13]. Intensification of land use, invasive species and pollution are driving both extinction and extirpation, altering the composition of local communities at an alarming rate [14,15]. Anthropogenic climate change is also shifting the ranges of species [16], and has the potential to significantly impact the geographical distribution of biomes in the near future [7], including risks of abrupt shifts [17]. These vegetation transitions will result in feedback through changed land cover, probably contributing to additional climate warming [18–20].

Modelling what the future distribution of vegetation types might look like will be fundamentally important for conservation planning and management, particularly if certain biomes—and the species within them—are to be protected [7,21–24]. In addition, half of the world's human population live in areas which are highly vulnerable to biome change [7]. Such perturbations have the potential to negatively impact human wellbeing, through alterations or reductions in the ecosystem services they produce or receive [7,25,26]. This could prompt changes to the locations and forms of settlements, and their associated political economies [27].

Previous attempts to model vegetation change into the future have generally ended their projections at 2100 (e.g. [5,7,19]). However, climate change is expected to extend well beyond this interval [28], and will continue to reconfigure global environments for centuries to come [27,29]. Here, we build on the approach taken in Lyon *et al*. [27], who modelled climate change between 2000 and 2500 CE using different representative concentration pathway (RCP) scenarios [28]. We combine HadCM3 global climate model outputs with BIOME4, a vegetation model [30], to infer how the area suited to different biomes will change over time under these climate scenarios. We use these projections to infer how biomes could be geographically distributed in the future, and the impact this might have on their total area over time. We then investigate the feasibility of vegetation migrating to these locations, taking into account potential overlap with human land use using the History Database of the global Environment (HYDE) anthromes [31].

## 2. Methods

### (a) Climate models

Projected future climate change was modelled under three different RCP scenarios: 2.6, 4.5 and 6.0 [32]. RCP2.6 represents a strong reduction in anthropogenic greenhouse gas emissions. RCP4.5 and RCP6 are medium stabilization scenarios, with moderate and limited levels of mitigation, respectively. RCP8.5 describes unmitigated growth in greenhouse gas emissions, but has been argued to be problematic and outdated [33]: this scenario would require the repeal of recent policy changes and commitments, and requires actions, such as a fivefold increase in coal burning by 2100, that have subsequently been demonstrated to be unrealistic [34]. We therefore chose to use RCP6 as the highest atmospheric greenhouse gas scenario.

Three simulations were run using the HadCM3 version of the UK Met Office Unified Model [35], each driven by atmospheric greenhouse gas concentrations from a different RCP scenario (2.6, 4.5 and 6.0), as outlined in Meinshausen *et al*. [28], using the procedure described in Lyon *et al*. [27]. The HadCM3B-M2.1a version of the model [36], used here, includes the top-down representation of interactive flora and foliage including dynamics (TRIFFID) dynamic global vegetation model (DGVM; [37]). This vegetation model simulates the fractional coverage of plant functional types (PFTs) within each climate model gridbox, based on a carbon balance approach, which actively updates the physical parameters in the land surface scheme. TRIFFID enables the climate model to incorporate many vegetation-climate feedbacks and outputs, as seen in Lyon *et al*. [27].

Each of these simulations were run from spun-up pre-industrial initial conditions at 1850 CE out to 2500 CE, with greenhouse gas concentrations fixed at 2300 CE levels from the extended RCP scenarios [28]. These simulations include the atmospheric greenhouse gas forcing of future climate change from carbon dioxide, methane and nitrogen dioxide, but not including other human-made pollutants, such as aerosols. Potential changes in ice sheets and sea-level rise, which could become important on these longer, multi-century timescales, are also not modelled. HadCM3 produces climate fields on a regular longitude-latitude grid of 3.75° × 2.5° (approx. 1.16 × 10^6^ km^2^ at the equator). The monthly mean temperature, precipitation and cloud amount fields were averaged over each 20 year period of the simulation from the most recent two decade period, 2000–2020 CE. These means were then downscaled with a standard anomaly method applied, from pre-industrial (1880–1899 CE mean) to observed reference fields [38] to produce suitable inputs for the BIOME4 vegetation model with a spatial resolution of 0.5° in latitude and longitude (approx. 3100 km^2^ at the equator). Downscaling to this extent provides a higher spatial resolution of climate variation, but without introducing unrealistic interpolation artefacts [30,39]. Higher-resolution climate datasets are beginning to be available (e.g. [40]), but these have yet to be fully tested with BIOME4.

### (b) Biome model

We converted the climate parameters from HadCM3 into projected climatic forcing on vegetation using BIOME4, a global vegetation model [30]. BIOME4 is a coupled carbon and water balance model that predicts the steady state, global vegetation distribution. It requires information on temperature, sunshine, precipitation, soil parameters (which are kept constant, as we do not model how they might change) and atmospheric CO_2_ concentration, for which we take the value at the midpoint of each 20 year period. The model maximizes the net primary productivity for each of 12 possible PFTs and then, based on a ranked combination of these PFTs, assigns each gridpoint to one of 26 biomes (table 1; [30]). The biomes inferred were also converted into nine ‘megabiomes' using the scheme of Harrison & Prentice [39]; this reduction in biome resolution was used to quantify more extreme projected floral transitions.

**Table 1.** The biomes [30], and the megabiomes to which they belong [39], as used in this study.

biome name	abbreviation	megabiome name
tropical evergreen broadleaf forest	TrEB	tropical forest
tropical semi-evergreen broadleaf forest	TrSB	tropical forest
tropical deciduous broadleaf forest and woodland	TrDB	tropical forest
temperate deciduous broadleaf forest	TeDBF	temperate forest
temperate evergreen needleleaf forest	TeENF	temperate forest
cool mixed forest	CoM	temperate forest
cool evergreen needleleaf forest	CoEN	temperate forest
cool-temperate evergreen needleleaf and mixed forest	CoTeEN	temperate forest
warm-temperate evergreen broadleaf and mixed forest	WTeEB	warm-temperate forest
cold evergreen needleleaf forest	CdEN	boreal forest
cold deciduous forest	CdD	boreal forest
tropical savannah	TrS	savannah and dry woodland
temperate sclerophyll woodland and shrubland	TeS	savannah and dry woodland
temperate deciduous broadleaf savannah	TeDBS	savannah and dry woodland
temperate evergreen needleleaf open woodland	TeENW	savannah and dry woodland
tropical xerophytic shrubland	TrX	grassland and dry shrubland
temperate xerophytic shrubland	TeX	grassland and dry shrubland
cold parkland	CdP	grassland and dry shrubland
tropical grassland	TrG	grassland and dry shrubland
temperate grassland	TeG	grassland and dry shrubland
desert	De	desert
graminoid and forb tundra	GFTu	dry tundra
low and high shrub tundra	ShTu	tundra
erect dwarf-shrub tundra	EDTu	tundra
prostrate dwarf-shrub tundra	PDTu	tundra
cushion-forb tundra	CFTu	tundra

HadCM3 and BIOME4 are commonly used together for modelling climate and biomes over longer-term scenarios, both in deep time and into the future (e.g. [41–44]). However, BIOME4 models biomes assuming that both climate and vegetation have reached an equilibrium state. Our input data represents the average simulated climate during each 20 year interval, and therefore does not capture any changes in climate within this time (although general trends over the total 500 years should be adequately captured). In addition, 20 years is probably insufficient time for vegetation to reach equilibrium in response to the modelled climate. As a result, our analyses describe climatic forcing on vegetation, but not necessarily realizable biome states, as transient ecosystem dynamics may be inadequately modelled.

### (c) Human footprint

All subsequent data cleaning, manipulation and analysis was carried out in *R,* version 4.2.2 [45] using the *geosphere* [46], *landscapemetrics* [47], *raster* [48] and *tidyverse* [49] packages.

In addition to our standard biome projections, we also conducted sensitivity analyses examining how interaction with human activity could impact biome realization. To do this, we created datasets in which we removed grid cells associated with human land use from our biome projections, under the assumption that human occupation could inhibit the migration of biomes into these cells. Human impact was assessed using the HYDE 3.2 anthromes dataset for the year 2017 [31]; in the absence of future projections, we assumed that these anthromes remained the same from the present day through to 2500. Two tiers of human influence were used: in the first, more relaxed tier, only ‘urban' and ‘dense settlement' environments were removed from the projections, while in the more stringent second tier, all environments except those classified as ‘wild' or ‘semi-natural' were removed.

The HYDE dataset also uses a latitude-longitude grid, but to a much higher resolution than our biome models (1/12° latitude and longitude). In order to compare the two datasets, the biome models were disaggregated to create smaller grid cells of the same size as those in the HYDE dataset. Cells with a human presence in the HYDE dataset could then be removed from the projected biome areas in each 20 year time slice.

### (d) Quantifying potential biome change

The nature of projected vegetation changes over time, between the three emissions scenarios, and using the different severities of human footprint, were quantified in five ways. Collectively, these statistics provide a summary of the distribution and coverage of area suited to each biome over time, until 2500. The area covered by each grid cell was calculated using the equation of Santini *et al*. [50], in order to convert biome projections for each grid cell into total terrestrial area.

Firstly, the biome projected to occupy each grid cell was compared between adjacent 20 year time slices, and with the present day (the 2000–2020 CE time slice). This was conducted both at a global scale and within low (30° N–30° S), mid (30°–60° N and S) and high (60°–90° N and S) latitude bands, to quantify the rate of change of climatic forcing over time, and the cumulative areal extent of climatic forcing towards a different biome compared to the present, respectively.

Secondly, the total area climatically suitable for each biome was calculated and compared between time slices. This indicates which biomes may gain total area over time under each scenario, and which biomes are more likely to lose area.

Thirdly, the proportion of area projected to be suitable for each biome within the same grid cells as in the previous time slice was calculated. One minus this value indicates the proportion of area which would require the colonization of a new region in order to be realized. As an extension of this, cells not adjacent to a cell of their projected biome in the previous time slice were also counted. These grid cells would require long-distance migration, or artificial transplantation, to successfully develop into their projected biome in that time slice.

Fourthly, the latitude–longitude coordinates of the centroid of each biome's projected area was found for each time slice. This was then used to determine the distance and bearing moved by each biome's centroid over time. This approach aimed to quantify the overall projected rate and direction of movement of the area suitable for each biome over time.

Finally, the number of patches (spatially segregated areas) suited to each biome was quantified using *landscapemetrics*::*get_patches*(), to determine the spatial cohesiveness of area suitable for each biome over time. This provides an indication of how easily migration, and genetic mixing, might occur between projected fragments of a single biome in each time slice.

## 3. Results

**Figure 1.**
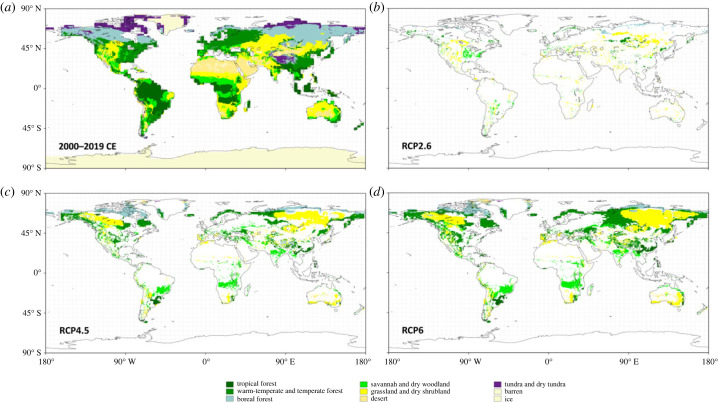
Maps of the area climatically suited to different megabiomes simulated with BIOME4, using the megabiome scheme of table 1. Present-day megabiomes are inferred using the mean climate of 2000–2019 CE (*a*). The other panels show the projected megabiomes in areas where a different megabiome is climatically suitable in 2480–2499 CE compared to the first time slice, under RCP2.6 (*b*), RCP4.5 (*c*) and RCP6 (*d*).

BIOME4 was used to infer climatic forcing towards different biomes across 1.31 × 10^8^ km^2^ of terrestrial area (all area which is ice-free and not described as ‘barren'; figure 1). Applying the HYDE dataset to this map increased the resolution of the coastlines, resulting in a reduced 1.25 × 10^8^ km^2^ of terrestrial area. Removing the area associated with more relaxed (urban) and more stringent (non-wild) anthromes left 1.24 × 10^8^ km^2^ and 6.18 × 10^7^ km^2^ of terrestrial area, respectively. The biome summaries given here were calculated as a proportion of these areal values.

**Figure 2.**
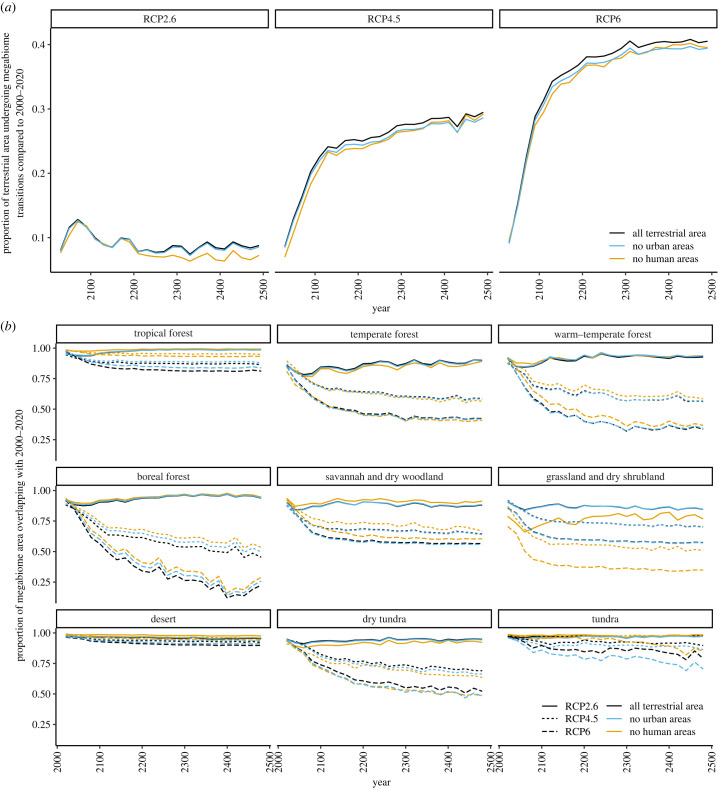
(*a*) For each RCP and human footprint, the amount of terrestrial area which is expected to change climatically suitable megabiome compared to the first time slice (2000–2020) over time. The area is quantified as all terrestrial land, all land except for urban conurbations, and only wild or semi-natural land. (*b*) For each megabiome and time slice, the proportion of climatically suitable terrestrial area which overlaps with that of the same megabiome in the first time slice (2000–2020).

**Figure 3.**
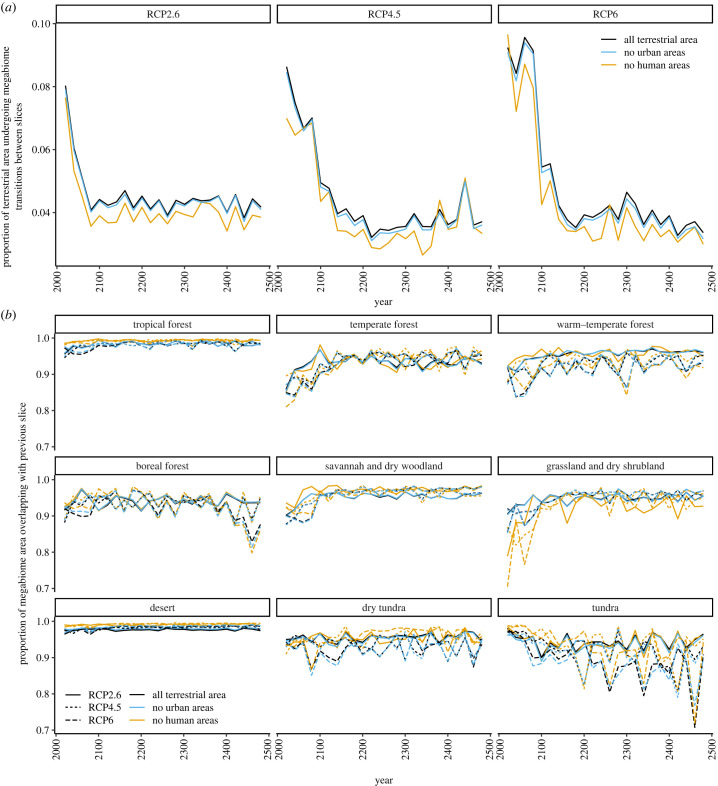
(*a*) For each RCP and human footprint, the amount of terrestrial area which is expected to change climatically suitable megabiome compared to the previous 20 year time slice. The area is quantified as all terrestrial land, all land except for urban conurbations, and only wild or semi-natural land. (*b*) For each megabiome and time slice, the proportion of climatically suitable terrestrial area which overlaps with that of the same megabiome in the previous time slice.

Even under RCP4.5, which is often considered a ‘business-as-usual' scenario, around 35% of terrestrial area is expected to be climatically suitable for a biome different to its present state, and 25% to a different megabiome, by 2150, with change continuing at a lower rate after this time (figure 2*a*; electronic supplementary material, S1a). This is matched by a high, but falling, rate of change between 20 year time slices from the present to 2150: 12% of land area is expected to experience climatic forcing towards a different biome per 20 years (and 7% to a different megabiome) by 2040, but by 2160, this falls to around 5%, which then remains consistent until 2500 (figure 3*a*; electronic supplementary material, S2a). The extent of human footprint applied does not substantially impact this result (figure 2*a*), although a slightly smaller proportion of wild and semi-natural regions is expected to experience climatic forcing towards a different biome over each 20 year period compared to all terrestrial environments (figure 3*a*). The mid- to high-latitudes are expected to experience more change, proportional to their area, than the low-latitudes (electronic supplementary material, figures S3a and S4a).

Under RCP6, similar but more extreme trends are seen to those of RCP4.5, with up to 50% of terrestrial area expected to experience climatic forcing towards a different biome than its present state, and 40% to a different megabiome, with this plateau only being reached between 2400 and 2500 (figure 2*a*; electronic supplementary material, S1a). By contrast, under RCP2.6, nearly 20% of land area is expected to be climatically suitable to a different biome, and 13% to a different megabiome, by 2080. However, some of this area will later revert, stabilizing at about 12% of terrestrial area being climatically forced away from its current biome, and 9% from its current megabiome, between 2250 and 2500 (figure 2*a*; electronic supplementary material, S1a). The rate at which area is climatically forced towards different biomes is also expected to fall faster under RCP2.6, reaching a plateau around 2100 (figure 3*a*; electronic supplementary material, S2a), but this plateau is at a higher rate than the other two emissions scenarios; while 5% of land area is expected to experience climatic forcing towards a different biome per 20 years by 2500 under RCP4.5 and RCP6, this is expected to be 6–7% under RCP2.6 (figure 3*a*; electronic supplementary material, S2a).

Unsurprisingly, it is biomes found in cold environments which are expected to lose the most total area of appropriate climate forcing. Area suitable for boreal forest biomes, both cold evergreen needleleaf forest and cold deciduous forest, is expected to decline under RCP4.5 and RCP6 (electronic supplementary material, figures S6 and S7). This is also true of cool mixed forest, although the area suited to temperate forest overall is predicted to increase. Tundra biomes are particularly hard hit, with prostrate dwarf-shrub tundra expected to have no climatically suitable area by 2160, and cushion-forb tundra predicted to completely lose climatically suitable area by 2300, under RCP6 (electronic supplementary material, figures S6 and S7). By contrast, grassland and dry shrubland biomes, including tropical and temperate xerophytic shrubland and tropical and temperate grassland, as well as desert, are expected to gain climatically suitable area over the next 500 years.

The proportion of overlap of area climatically suited to each individual biome, both with its present-day distribution and its distribution in the previous time slice, varies between biomes and over time (figures 2*b* and 3*b*; electronic supplementary material, S1b and S2b). For most biomes and megabiomes, the proportion of overlap is reduced at higher RCPs, implying a larger extent of loss or movement of suitable environmental conditions for that biome. For example, large swathes of area suitable for cool evergreen needleleaf forest are expected to shift even under RCP2.6, with only around 50% of its projected distribution in 500 years overlapping with its present distribution (electronic supplementary material, figure S1b). At higher RCPs, wholly different areas are expected to be climatically suitable for this biome in as little as 100 years. By contrast, the area suited to desert is projected to mostly maintain its present distribution throughout the next 500 years, with little change expected between 20 year time slices (figures 2*b* and 3*b*). Some biomes are projected to have abundant climatically suitable area that is not even adjacent to that in the previous 20 year interval (electronic supplementary material, figure S5). For example, under RCP4.5 and 6, up to 50% of the projected area suited to cool-temperate evergreen needleleaf and mixed forest in each time slice may not be adjacent to that in the previous 20 years.

Under RCP4.5, the latitudinal ranges of area suited to each biome in each hemisphere are expected to remain largely consistent over the next 500 years (electronic supplementary material, figure S8a). For most biomes, this includes no clear poleward shift in distributions, also confirmed by the bearing of movement of centroids of the area suited to each biome over time, which generally show no clear pattern (electronic supplementary material, tables S1-S3). There is also no clear trend in how connected the area suited to each biome is expected to be, either through time or across RCPs (electronic supplementary material, figure S9). However, many biomes do show evidence of strong fluctuation in the latitudinal ranges of their suitable area between adjacent time slices, such as temperate sclerophyll woodland and shrubland and temperate grassland (electronic supplementary material, figure S8a). For several biomes, including cold evergreen needleleaf forest, cold deciduous forest and low and high shrub tundra, the latitudinal ranges of their suitable area in the Southern Hemisphere are expected to strongly reduce.

## 4. Discussion

Our climate models indicate that under a ‘business-as-usual' scenario, climate change in response to anthropogenic emissions is expected to continue well beyond 2100 [27]. Here, we demonstrate that this may also result in substantial changes in the amount and distribution of area suited to different biomes, continuing long beyond the end of this century. Our RCP4.5 models show that up to 40% of terrestrial area will no longer be climatically suitable for its present biome by 2500 (figure 2*a*), with many cold-adapted biomes, particularly boreal forest and dry tundra, losing a large proportion of their suitable area (figure 2*b*). A higher proportion of area in the mid- to high-latitudes is expected to experience change than the low-latitudes (electronic supplementary material, figures S3a and S4a), particularly in the Southern Hemisphere (electronic supplementary material, figure S8a).

Our results show broad agreement with previous, similar analyses of changing biomes conducted on shorter timescales. Huntley *et al*. [5] predicted that 52% of terrestrial land will have changed biome under RCP4.5 by 2100, slightly higher than our estimate of 35%. Across the three RCPs, we show that between 12% and 50% of land area will be suited to a different biome in the year 2500 compared to the present-day; this is comparable to the range estimated by Gonzalez *et al*. [7], who used a variety of climate models and DGVMs to infer that between one-tenth and one-half of global land area may currently be highly vulnerable to biome change.

Even under RCP2.6, we expect 6–7% of land area to become climatically suited to a different biome every 20 years from 2100 onwards (figure 3*a*). This is owing to fluctuations in the area suitable to some biomes, which can also be seen in their expected latitudinal distributions (figure 3*b*; electronic supplementary material, S8a). Such fluctuation has previously been recognized in studies of present biome distributions: for example, Friedl *et al*. [51] estimated that around 10% of terrestrial area currently undergoes biome transition per year. Our RCP4.5 and RCP6 projections reach a slightly lower (5%) plateau in the rate of becoming suitable to a different biome after 2200 (figure 3*a*), however in these cases, it is associated with continued divergence from the biome distribution of the present-day (figure 2*a*). This suggests that it will take longer to reach a constant rate of change under these scenarios, and that once reached, they may have a lower rate of natural fluctuation compared to the RCP 2.6 scenario, and the recent past.

In most of our analyses, the impact of removing the human footprint on the proportional loss of area suited to each biome was minimal, particularly in comparison with the difference between emissions scenarios (e.g. figure 2*a*). This was also true for Higgins *et al*. [2]. However, wild and semi-natural regions were projected to experience a slightly lower rate of change over time than all terrestrial environments collectively (figure 3*a*). This difference was most pronounced at low-latitudes (electronic supplementary material, figures S3b and S4b). This finding indicates that proportionally more change might be expected to take place within regions which are currently within the human footprint, particularly agricultural land. However, we modelled anthromes as being static over the next 500 years, and human impacts on the environment have increased in area and severity over the last 30 years [52], and are expected to continue to do so. As such, it is likely that land use change will become an increasingly important factor in limiting biome migration in future.

Important questions arise as to whether the biome changes projected here are ecologically possible. We describe patches of terrestrial area which would be climatically amenable to a particular biome, but there are many barriers to these patches being realized, including limited dispersal, geographical and anthropogenic barriers, and competitive exclusion [7]. Dispersal rates, in particular, are a major concern given that we expect many patches to arise a substantial distance from the area previously suited to that biome (electronic supplementary material, figure S5; [53]). Tree migration rates are estimated to be only 100 m to 10 km per year [54,55], and many boreal and temperate tree species may only be able to migrate to between a quarter and half of their newly arising suitable habitat over the next century [56–58]. However, addressing questions about migration feasibility in our models is difficult, as dispersal ability is highly heterogeneous between different species within a biome, compounded by local variability in climate change and terrain [59]. Regardless, it is clear that for some biomes, we infer that suitable climatic conditions will arise in regions too far from their prior distributions to reach by migration. Human intervention serves as a potential solution: relocating species to more amenable habitat as a means of conserving them, or to provide benefits to humans, is a key action proposed for climate adaptation and practised today [60,61]. However, this must be carefully planned, particularly given the uncertainties and unintended consequences of introducing non-historically native species into new ecosystems [61,62].

Even if dispersal to a newly arising area of suitable climatic conditions is possible, there are questions about how the process of transition takes place, and the time needed for a new biome to establish (e.g. [63]). Functional convergence of the plants within a patch towards a particular biome is typically assumed to be owing to environmental filtering of those poorly adapted to those conditions, and selection of those which are well adapted [3]; these are processes which probably require more time than the 20 year intervals we model here. In some cases, it is possible that the biome changes we predict would not take place. Biome delineation is both less exact and more complex than our models suggest, with some biomes occupying a range of environmental conditions, and groupings of environmental conditions being associated with multiple biomes [2,3]. It is also not well understood how historical contingency can play a role in determining biomes [3,11,64]. Further, disturbances such as herbivorous grazing and fires can 'consume' existing vegetation, accelerating the realization of a new biome [3], while in some cases, anthropogenic changes in fire regimes, whether via hunting-gathering or agriculture, have been found to increase or maintain open woodland and savannah biomes [65]. Gaining more knowledge on how biome transitions take place, and the timescales on which they occur, is an important next step in validating our model projections.

Large areas of land transitioning biomes over the next 500 years would have wide-reaching impacts on both biodiversity and society. The projected reduction in suitable area of some biomes, if realized, would lead to extinction for many plants adapted to those environments (e.g. [66,67]). As climatic conditions become unsuitable for the survival of current vegetation, a reduction of plant biomass and terrestrial carbon storage is expected, either gradually or through sudden, destructive events such as wildfires [5,7,19,20]. Biome stability is also strongly correlated with regional biodiversity, indicating that more recently perturbed regions are less diverse [5,68]. Faunal biodiversity will face the double threat of changing climate and the loss or migration of vegetation on which it relies, and will need to either migrate in concert or adapt to survive [69].

As biomes change, the stocks and flows of ecosystem services they provide will come into question. These include services fundamental to human survival and habitability, such as food and water [70]. They also include protecting human populations against the increasing frequency and severity of various weather events; for example, tropical mangrove forests reduce the severity of storm surges [71]. Such events might otherwise require adaptations that may face significant social, technological, and physiological challenges or limits, or could even force human relocations [72,73]. However, as some regions become less habitable, others may open [73], and society must develop plausible, desirable and equitable strategies to share landscapes, access ecosystem services, and avoid conflict while learning to live with these changes [74,75].

Our results agree with previous studies that boreal and tundra biomes, which are mostly found at mid- to high-latitudes, are likely to lose a considerable proportion of their climatically suitable area over the next few centuries (figures 2*b* and 3*b*) [2,7,19]. The boreal biome provides a number of ecosystem services, including timber forest products, amenities and carbon sequestration, which stand to become depleted as the biome wanes. It is also highly prone to disturbances from fire and pests [76,77], including recent record wildfires that are projected to intensify [77–79] as higher latitudes warm much faster than the global average, threatening regions of the biome that remain. Increased efforts should therefore be made to protect boreal and tundra biomes specifically, but this will need to take into account the geographical shifts these biomes may make over the next 500 years.

This study presents, to our knowledge, the first simulations of potential climate-driven biome changes to 2500 CE and, as such, has used available climate model simulations and an equilibrium biome model often associated with this general circulation model. Future climate and biodiversity are highly uncertain, and the testing of additional models, both for climate and vegetation, would be beneficial, ideally at a higher spatial and temporal resolution [7,57,80]. Our climate modelling approach used TRIFFID, which allows interaction between the vegetation and local climate to be taken into account [3], but mismatches between the vegetation inferred by TRIFFID and the biome projections produced using BIOME4 are possible (e.g. [11]). The use of a DVGM to infer biomes, rather than an equilibrium model, would also allow migration to be simulated within the model (e.g. [81]). Terrestrial ice area was fixed, so the greening of currently frozen land could not be taken into account, and corresponding sea-level rise was not modelled. Our climate models use cells delineated by degrees, which vary considerably in their surface area, and this means the spatial resolution of our results are variable with latitude. Our use of a static human footprint for the year 2017 [31] is also suboptimal, as the space occupied by anthropogenic activities is likely to increase in future [52]. However, the exact geography of this expansion is difficult to predict. While we consider anthropogenic greenhouse gas emissions to represent the biggest source of uncertainty in our results, investigation of these other factors may reveal more insights to future biome change which could prove useful in planning for mitigation and adaptation.

## 5. Conclusion

By modelling how climatic forcing towards different biomes may manifest in future, our research contributes to a new but growing body of work examining climate change impacts after 2100. We show that across climate scenarios, significant changes in the global distribution and areal extent of land amenable to different biomes will probably occur, particularly over the next two centuries. Cold-adapted biomes are expected to lose the greatest amount of climatically suitable area, particularly impacting mid- to high-latitude regions. This will probably have strong implications for biodiversity, and the ecosystem services that humans rely on. Our results highlight the necessity of better understanding climate and biome change past 2100, in order to mitigate and adapt to the many challenges these changes will present for ecosystems and humanity.

## Data Availability

The data and code are provided in the Zenodo repository: https://doi.org/10.5281/zenodo.10820536 [82], and the supplementary figures in the electronic supplementary material [83].
